# Targeting Intracellular miRNA in Different Cancer Cell Models Using Gold Nanoprobes and Combined Mass Cytometry and Single Particle ICP-MS

**DOI:** 10.1021/acs.nanolett.5c02886

**Published:** 2025-07-15

**Authors:** Sara González-Morales, Lena Schlautmann, Paula Díez, Jörg Bettmer, Mario Corte-Rodríguez, Maria Montes-Bayón

**Affiliations:** † Department of Physical and Analytical Chemistry, Faculty of Chemistry, University of Oviedo, Julián Clavería 8, 33006 Oviedo, Spain; ‡ Health Research Institute of the Principality of Asturias (ISPA), Avenida Hospital Universitario s/n, 33011 Oviedo, Spain; § Institute of Inorganic and Analytical Chemistry, University of Münster, Corrensstrasse 48, 48149 Münster, Germany; ∥ Department of Functional Biology, Immunology Area, Faculty of Medicine and Health Sciences, University of Oviedo, Health Research Institute of the Principality of Asturias (ISPA), Avenida Hospital Universitario s/n, 33011 Oviedo. Spain

**Keywords:** miRNA, mass cytometry (CyTOF), SC-ICP-MS, gold nanoprobes

## Abstract

DNA-conjugated gold nanoparticles (AuNPs) were developed to target intracellular miRNA-16-5p across various cancer cell models by base pair complementarity. The Au-nanoprobe uptake was addressed by multiparametric mass cytometry (CyTOF) monitoring iridium and gold, enabling discrimination among Au nanoprobes in intact cells and cellular debris. Our findings reveal significantly higher incorporation in lung cancer (A549) and melanoma (A375) cells compared to hepatic (HepG2) and ovarian (A2780) models with particle numbers ranging from 200 to 1 AuNPs per cell, respectively. The internalized Au nanoprobes targeting miR-16-5p were captured by mixing the lysed cells with a half-complementary DNA probe immobilized on streptavidin-coated magnetic microparticles. By counting the Au events in the captured solution is possible to quantitatively assess the concentration of miR-16-5p on each cell line. Together, these two complementary MS-based strategies establish a platform for the quantitative evaluation of nanocarrier-mediated miRNA targeting, offering new avenues for the development of miRNA-based cancer therapeutics.

## Graphical abstract

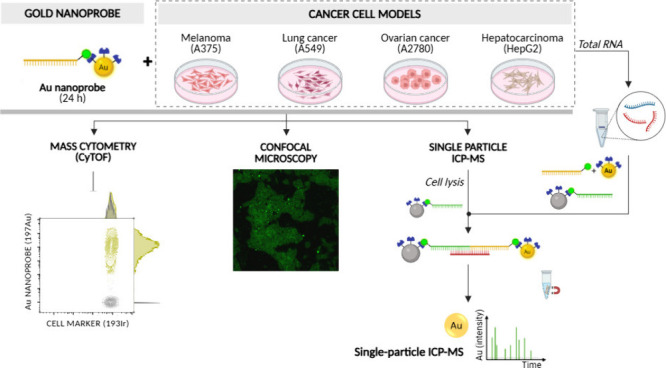

Microribonucleic acids (miRNAs) are small noncoding RNA biomolecules (18–24 nucleotides) that regulate gene expression by binding to complementary sequences in mRNA (mRNA), enabling post-transcriptional silencing or degradation.^1,2^ Since their discovery, they have been the center of biomedical research, particularly for miRNA-therapeutics for which miRNAs should be targeted (e.g., for miRNA inhibition using antisense oligonucleotides).^3,4^ However, sustained delivery of these therapeutic agents remains challenging due to the limited cellular uptake of nucleotides and their instability, making them prone to be easily degraded in the biological systems.^5^ In this context, oligonucleotide-conjugated nanoparticles and in particular, gold nanoparticles (e.g., DNA-AuNPs) represent an interesting alternative that has been explored by some authors.^6,7^ Nevertheless, before the clinical use of such therapies, several aspects have to be addressed by the development of adequate bioanalytical platforms. First, those aspects related to the quantitative delivery efficiency of the Au-DNA nanoconjugates into different cell types and second, the intracellular fate of the nanoconjugates,^8^ in particular, their capabilities to specifically target intracellular miRNA sequences with adequate selectivity. The cellular incorporation of Au-DNA nanoconjugates has been estimated in some cases by using inductively coupled plasma-mass spectrometry-based methods (ICP-MS) in “bulk” mode, this is, by analyzing the total metal concentration in lysed counted cell population.^9^ However, this approximation neglects cell heterogeneity, averaging the cellular metal incorporation of the probe into the exposed total cell number concentration. Therefore, knowledge of the nature of the cell incorporation and potential damage caused by the Au-DNA nanoconjugate at a single-cell level is essential for gaining a more in-depth understanding of cell–NP interactions.^10^ In this regard, the use of single-cell (SC-ICP-MS) approaches has permitted elemental information on individual metal content per cell using specific sample introduction systems and sample preparation strategies.^11–13^ Mass cytometry (a.k.a. CyTOF), commercially available since 2010, represents an addition to the SC-ICP-MS-based technology that allows simultaneous detection of multiple elements in single particles or cells due to the fast scanning capabilities of the time-of-flight (Tof) mass analyzer.^14,15^ This technology possesses the capabilities to provide simultaneous information on the free and cell-associated Au nanoprobes, as well as on the cell debris, using adequate cell markers.^16,17^ In addition, immobilizing on the AuNP a complementary sequence to a mature cytosolic miRNA should allow the evaluation of the intracellular targeting process by combining an additional cell lysis experiment without involving tedious nucleic acid amplification reactions.^18,19^

Here we have developed an MS-based single-cell/single-particle bioanalytical platform to gain in-depth knowledge about the intracellular trafficking of Au-DNA nanoconjugates as well as their capacity to specifically target cytosolic miRNA. First, the cellular incorporation of the nanoconjugates was investigated to establish the between- and within-population cell heterogeneity using CyTOF to simultaneously detect the nanoconjugates and an iridium DNA labeling compound to distinguish the internalized nanoconjugates.^20,21^ Second, the potential of the incorporated Au nanoconjugates for targeting and quantifying an intracellular miRNA, without amplification reactions was explored by parallel use of SC-ICP-MS. For this purpose, the selected oligonucleotide used for AuNP conjugation was a DNA-complementary sequence to a well-studied miRNA, miR-16-5p, which is involved in different carcinogenic processes.^22^ All the studies were performed in different cancer cell models (ovarian, lung, skin and hepatic malignant tumors) for which different levels of expression of miR-16-5p are expected.^23^ The general scheme of the bioanalytical platform is graphically detailed in Figure 1.

**Figure 1:**
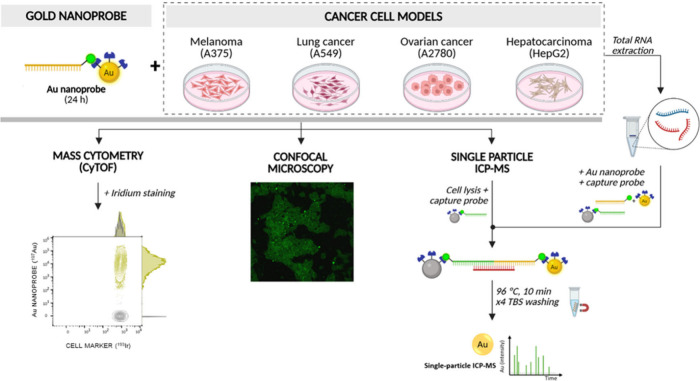
Scheme of the combined strategy used for the elucidation of the cell–Au-nanoprobe interactions (including cellular incorporation) and the capturing/quantification of the intracellular miRNA. [Created with Biorender.com.]

Polyethylene glycol (PEG) coated gold 40 nm nanoparticles labeled with a DNA sequence that is half-complementary to the miR-16-5p (see the Supporting Information Table S1) were used as recognition probes (synthesis and characterization as described in the Supporting Information and Figure S1). Four different tumoral cell lines from different origins (A375 melanoma, A549 lung cancer, HepG2 hepatic cancer and A2780 ovarian cancer) were exposed to 20 μM Au-DNA nanoprobes for 24 h. Cells were then trypsinized and analyzed by fluorescence microscopy (taking advantage of the surface plasmon resonance (SPR) effect of gold nanoparticles). After the treatment, the presence of the Au nanoprobes inside the cells was observed, as shown for HepG2 cells in Figure 2A (yellow arrows) but also in the extracellular space either alone and/or associated with cell debris (blue arrows). Such debris can be generated as a consequence of the toxicity associated with the exposure to the Au nanoprobe or the cell trypsinization process. To obtain absolute quantitative information regarding the content of extracellular Au nanoprobes, Au nanoprobes associated with cells and cell debris, cells were fixed, washed and labeled with an iridium (Ir) DNA intercalator used as cell marker. Simultaneous elemental signatures of Au and Ir were taken using CyTOF (gating strategy shown in Figure S2) and can be observed in Figure 2B–E, plotting the Ir (*X*-axis) versus the gold signals (*Y*-axis) for the A375, A549, A2780, and HepG2 cells, respectively. The different color-coded distributions located at the upper-right corner of each diagram correspond to gold-containing intact cells (Au^+^/Ir^+^). The gray distributions on each diagram correspond to the intact cells (Ir^+^) without detectable gold signal (Au^–^), which belong to cells that did not incorporate any Au nanoprobe.

**Figure 2:**
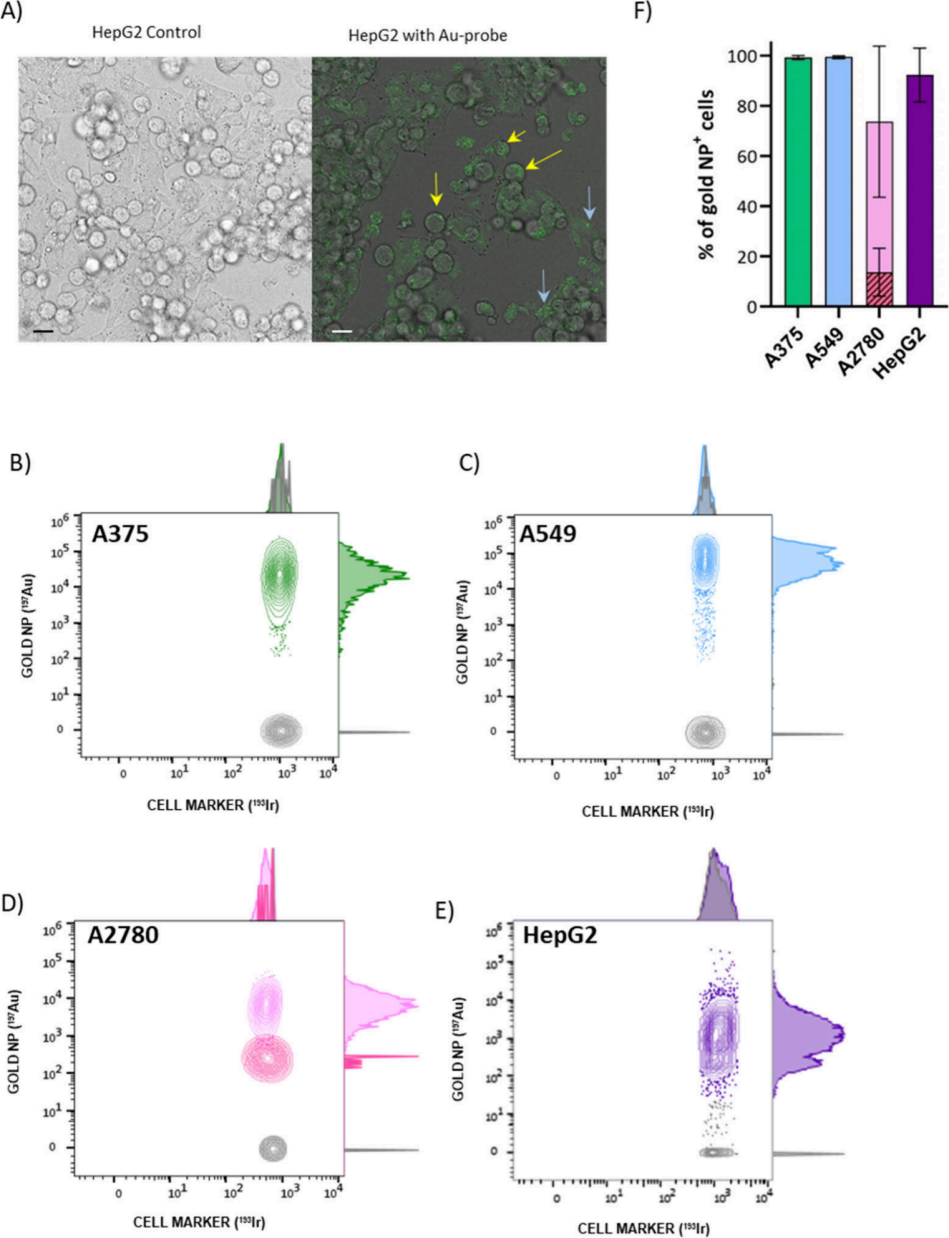
(A) Micrographs showing HepG2 untreated cells (left) and the same cells treated with the AuNP-DNA probes for 24 h. Yellow arrows point to internalized nanoparticles and blue arrows point to nanoparticles in the extracellular space. Scale bar is 20 μm. Contour 2D plots Ir and Au signals for (B) A375, (C) A549, (D) A2780, and (E) HepG2 cell lines treated with AuNP-DNA probes. (F) Differential behavior among the different cell lines regarding AuNP incorporation.

For quantitative analysis, Au^+^/Ir^+^ cells were counted across areas of intact cells by using the gating strategy described (Supporting Information Figure S2). In summary, a differential behavior among cell lines can be observed (Figure 2F) revealing that the intact cells that did not capture any Au nanoprobe (gray distributions) represented only about 2% of the total in the cases of A375 and A549 cells followed by HepG2 (10%). A2780 cell line showed a heterogeneous profile with two populations presenting different levels of Au-nanoprobe incorporation, for a total of 70% intact cells containing particles. Thus, this cell model showed a significantly lower level of Au-nanoprobe incorporation, revealing that 30% of the intact cells did not contain any detectable Au signal. However, the unique capabilities of the multielemental simultaneous measurements of CyTOF permit to obtain further information regarding the presence of other species coexisting within the cell suspension (e.g., cell debris or unbound AuNPs). For this aim, all events were plotted in an Ir vs Au plot to visualize the overall distribution (Figure 3).

**Figure 3:**
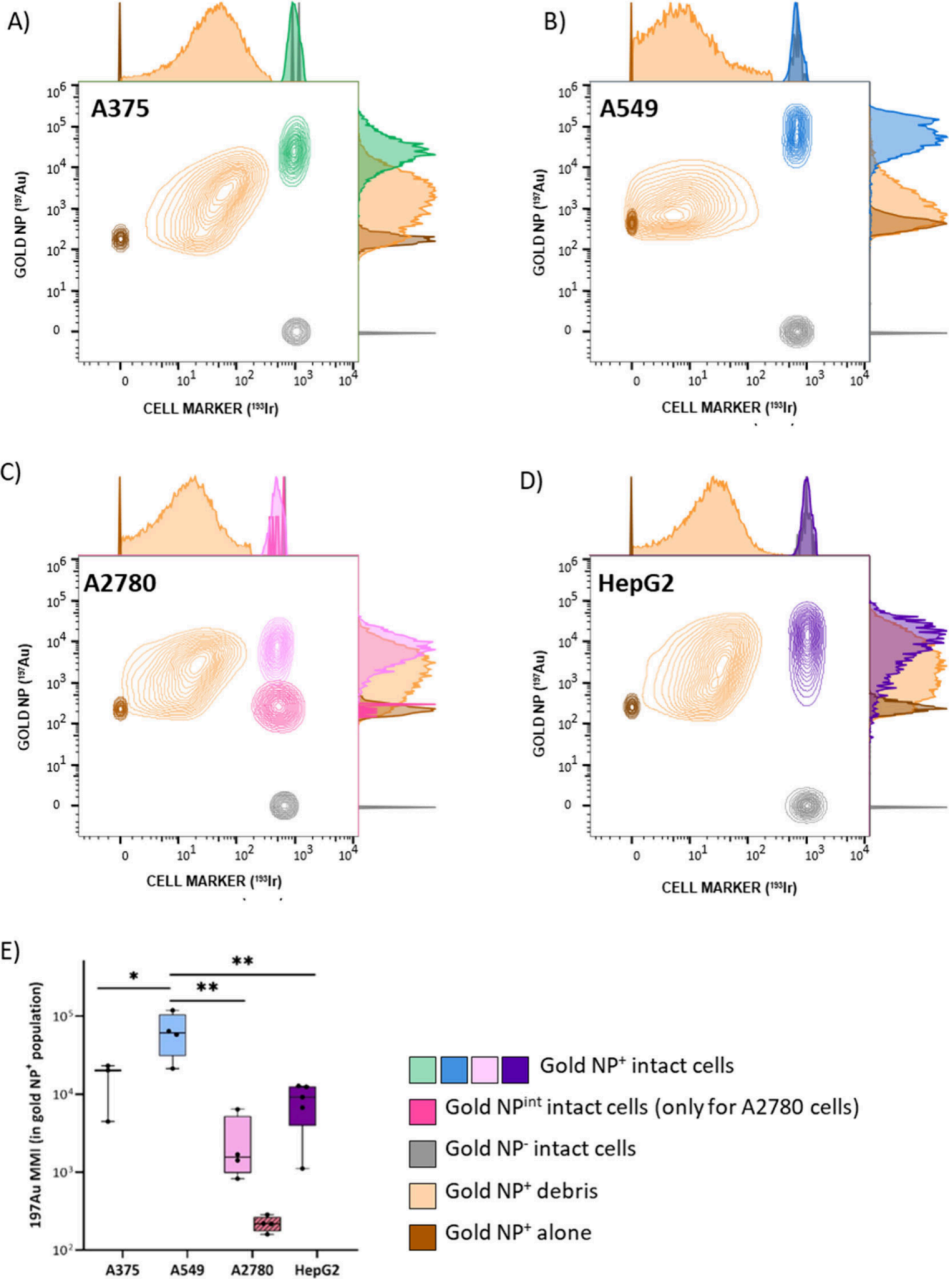
Contour 2D plots showing the Au and Ir content in events, including those not corresponding to cell populations in samples. Color coded populations corresponding to (A) A375 (green), (B) A549 (blue), (C) A2780 (pink), and (D) HepG2 (purple). Dark brown populations correspond to free Au nanoprobes. Orange populations correspond to cell debris. Gray populations are intact cells that have not incorporated Au. (E) Au-nanoprobe incorporation in all cell lines as nanoparticles per cell. MMI, median metal intensity.

Here, two additional populations were detected on top of those shown in Figure 2. One population containing gold but not iridium (Au^+^/Ir^–^) (dark brown population in the plots of Figure 3A–D) ascribed to free Au nanoprobes remaining in solution, as a result of weakly adsorbed particles to the outer cell membrane and released during sample preparation. These events do not represent a very dense population in any of the plots but show similar intensities in all of them for the different cell lines. Therefore, it supports the fact that they correspond to the same species. Confirmation regarding the nature of these species was obtained by analyzing a suspension of Au nanoparticles of the same diameter measured under the same conditions (see Figure S3).

A second large and dispersed population (wide distribution in orange in the plots of Figure 3A–D) corresponds to a variable content of Au and Ir (Au^dim^/Ir^dim^) and is ascribed to the presence of cell debris. This type of cell debris, observed to a different extent for all the cell models under evaluation (see Figure 3A–D) can be ascribed, at least partially, to the cell damage originated by the endocytic-mediated incorporation of large numbers of Au nanoprobes into cells.^24^ In this regard, the comparative levels of probe uptake among intact cells for the four different cancer models can be observed in Figure 3E. Based on these data, cell models with higher levels of Au-nanoprobe incorporation (A375, with Au median intensities around 3 × 10^4^ dual counts (arbitrary signal intensity units in CyTOF. These values correspond to the integrated intensity across all data points forming a cell event) and A549, with Au median intensities around 8 × 10^4^ dual counts) showed a population of Au^+^ intact cells that represents 17% and 23%, respectively, out of the total Au^+^ events. Contrarily, models with lower levels of nanoprobe incorporation (HepG2 and A2780 with median Au intensities around 10^4^ and 10^2^–10^3^ dual counts, respectively) contain Au^+^ events associated with intact cells representing 44% and 52%, respectively. Therefore, an inverse correlation can be established between the level of incorporation of the Au nanoprobe in the intact cells and the generation of cell debris, statistically significant among all of them (see Figure 3E).

By comparing the gold intensities achieved for the “free” Au nanoprobes on each plot with the gold intensities obtained in the individual cells is possible to estimate levels of incorporation of about ∼200 AuNPs/cell (A549), ∼120 AuNPs/cell (A375), ∼24 AuNPs/cell (HepG2), and between 1 and 4 AuNPs/cell (A2780, considering the two cell populations observed). The differential uptake of 40 nm nanoparticles across cancer cell lines—highest in lung cancer, followed by melanoma, liver carcinoma, and ovarian cancer—might stem from variations in endocytic activity, membrane properties, receptor expression, and metabolic demands. Thus, these lung cancer cells seem to exhibit enhanced clathrin-mediated endocytosis^25,26^ and abundant surface receptors (directly or via protein adsorption), driving efficient internalization via these mechanisms. On the other hand, melanoma and liver carcinoma cells show reduced endocytic activities or receptor availabilities, compared to lung cancer cells, potentially tied to metastatic adaptations. Finally, ovarian cancer cells display the lowest internalization, likely due to fewer receptors, altered membrane composition, or suppressed endocytic pathways.^27^

Regarding nanoparticle aggregation, all cell-growing media had been enriched with the same amount of fetal bovine serum, therefore similar aggregation of the Au nanoprobe is expected. Additionally, the added Au nanoprobes contain a half-complementary sequence to miR-16-5p. It is known that mature miRNAs function intracellularly either free or as part of a larger ribonucleoprotein complex where they provide transcript specificity. The binding of miR-16-5p to the internalized Au nanoprobe may alter the trafficking of the nanoparticles, leading to greater retention inside the cells, particularly in the cytoplasm where mature miRNAs are processed and most of them carry out their function.

Thus, to further test the capabilities of the incorporated Au probes to specifically target intracellular miR-16-5p, we used a modification of the developed analytical approach that was previously tested in cell lysates.^28^ For this aim, the cells containing the internalized Au nanoprobes were lysed and then treated with a capture probe (half complementary sequence to the target miRNA functionalizing a magnetic bead by streptavidin–biotin interaction) to create a double hybrid with the target (see Figure 1). The complex, containing the Au nanoprobe, the target miRNA and the capture magnetic probe, was enriched and purified by using a magnet. Once captured and washed, the double hybrid was disassembled by heating the mixture to 97 °C for 10 min, causing the denaturation of all nucleic acid hybrids, as well as the biotin–streptavidin interactions. This process separated the gold nanoparticles from the magnetic beads that could be magnetically removed. The resulting gold nanoparticle suspension was then adequately diluted and measured by single particle ICP-MS (SP-ICP-MS) allowing the counting of the particle number concentration in the final solution. In this case, the number of particles can be directly related to the number of miR-16-5p molecules in solution by conducting a calibration curve using standard solutions of the sought analyte and provided that the transport efficiency in both cases is comparable. Obtained results for the determination of miR-16-5p in the four cell lines under study are shown in Figure 4A. First, these results revealed that the Au nanoprobe was able to target the intracellular miR-16-5p and the magnetic bead-immobilized probe was also able to capture the hybrid in a quantifiable form. By comparing among cell models, A375 (melanoma) showed a higher concentration of miR-16-5p than all the other cell lines, followed by A549 and HepG2. Significantly, lower concentrations were observed in A2780. This is in agreement with databases reporting the level of expression of miRNAs (relative) in cell models following the sequence A375 > A549 > HepG2 > A270 (see Figure S4).^23^

**Figure 4:**
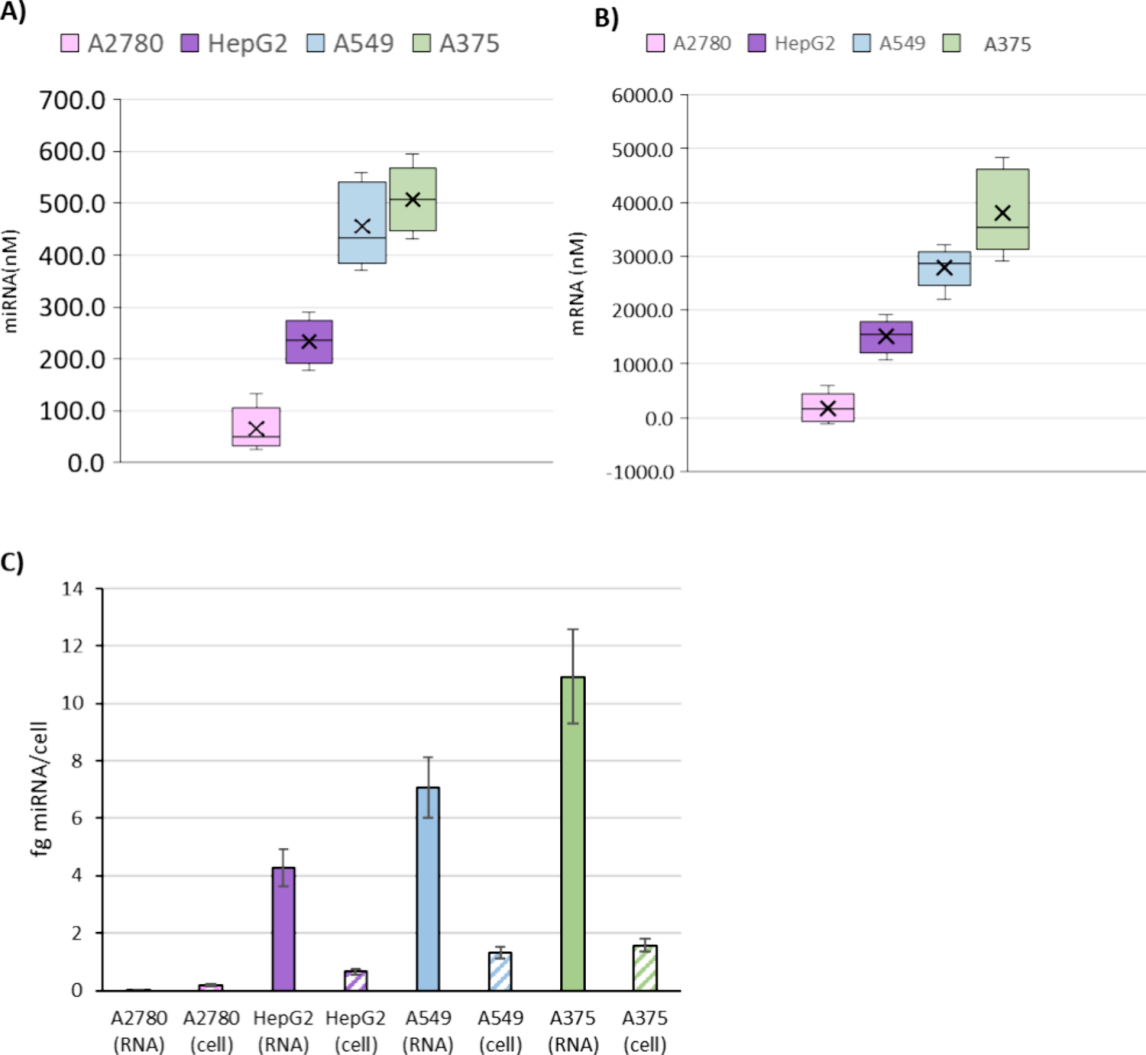
Obtained miRNA concentrations in the different cell lines by (A) assay based on incorporated nanoparticles (not lysed) and (B) hybridization assay after cell lysis and miRNA extraction. (C) Comparison of both methods, referred to as fg miRNA/cell.

In a parallel experiment, cell batches of each nontreated cell model were subjected to cell lysis and total RNA extraction (including miRNA) using the standard TRIzol protocol.^29^ The assay for the miRNA quantification was then performed on the purified RNA by adding the Au nanoprobe followed by the magnetic probe for capturing the hybrid. In this case, after the detection and capture probes were added, the mixture was incubated at 70 °C for 10 min to denature any hybridization or secondary structures of the probes or the analyte. This temperature was higher than the melting point of all the oligonucleotides used, but lower than 80 °C, which could cause the denaturation of biotin. The mixture was left to cool down slowly until room temperature to guarantee specific hybridization and then washed. The obtained results for miRNA16-5p quantification in the four cell lines under evaluation can be also seen in Figure 4B. A similar trend can be observed by comparing the results of Figure 4A,B for the four cell models. The absolute concentration levels are, however, consistently higher in the case that the assay is conducted over the extracted RNA.^30^ This can be also comparatively seen in Figure 4C where the results are plotted as fg miRNA/cell using the two strategies. These differences are approximately 6-fold in the case that the miRNA concentrations are determined over extracted RNA (solid bar) with respect to the whole cell (dashed bar) in all cases. One plausible explanation for these differences could be that the level of incorporation of the Au nanoprobe is not high enough to target the whole miRNA present within the cell cytosol. It should be considered that there is a cellular exocytosis mechanism that might act to eliminate some of the incorporated probes into the extracellular space. In addition, it could also be due to the presence of the sought sequence in a larger ribonucleoprotein complex that cannot be either targeted by the probe or captured by the magnetic probe.

Altogether, we show that the combined use of CyTOF and mass spectrometry-customized experiments allow the quantification of the level of incorporation of Au nanoprobes into cells. This can be extremely advantageous for addressing the fate of nanotransported therapies and in particular for miRNA therapeutics for which miRNAs should be targeted. In this regard, the Au nanoprobe used to target miRNA16-5p has proved to be efficiently incorporated in most cell models under study but has revealed significant differences among them. Furthermore, the incorporated Au nanoprobe has been able to bind, specifically, to the miRNA-16-5p present in the cell cytosol targeting this molecule whose concentration could be obtained directly using the Au label using our novel strategy. Our study provides also an example of the complementary use of mass cytometry and mass spectrometry to gain in-depth knowledge about the intracellular trafficking of gold nanoparticles as well as their capacity to specifically target cytosolic miRNA and can be incorporated into future assessments on the use of nanotransported miRNA inhibitors, for instance, as therapeutic agents. Further studies to explore the multiplexing of the assay by binding different oligos to other types of NPs could be the next steps to take.

## Supplementary Material


