# Supplementary material for: Targeting Intracellular miRNA in Different Cancer Cell Models Using Gold Nanoprobes and Combined Mass Cytometry and Single Particle ICP-MS
Source: Nano Lett. 2025 Jul 15;25(29):11492–8. doi: 10.1021/acs.nanolett.5c02886 (PMC12291581; doi:10.1021/acs.nanolett.5c02886)
Supplement: Supplementary file 1 [file nl5c02886_si_001.pdf]

SUPPLEMENTARY INFORMATION

TARGETING INTRACELLULAR miRNA IN DIFFERENT CANCER CELL MODELS USING GOLD NANOPROBES AND COMBINED MASS CYTOMETRY AND SINGLE PARTICLE ICP-MS.

Sara González-Morales[†], Lena Schlautmann[‡], Paula Díez[‡,ª], Jörg Bettmer[†], Mario Corte-Rodríguez[†]* and Maria Montes-Bayón[†]*

[†]Department of Physical and Analytical Chemistry, Faculty of Chemistry, University of Oviedo, Julián Clavería 8, 33006 Oviedo, Spain. Health Research Institute of the Principality of Asturias (ISPA), Av. Hospital Universitario s/n, 33011 Oviedo. Spain.

[‡]Institute of Inorganic and Analytical Chemistry. University of Münster. Corrensstr. 48, 48149 Münster, Germany.

[‡,ª]Department of Functional Biology. Immunology Area. Faculty of Medicine and Health Sciences, University of Oviedo. Health Research Institute of the Principality of Asturias (ISPA), Av. Hospital Universitario s/n, 33011 Oviedo. Spain.

*montesmaria@uniovi.es; cortemario@uniovi.es

Instrumentation

The measurements were performed using the iCAP TQ triple quadrupole ICP-MS system from Thermo Fisher Scientific, Bremen, Germany. All measurements, including single particle ICP-MS measurements, were done using the standard configuration for sample introduction, including the Micro-Mist nebulizer at a sample flow rate of 0.4 mL min^{-1} and a cyclonic spray chamber. The sample introduction set-up was used together with a Teledyne Cetac ASX-560 autosampler. Daily optimization of the ICP-MS was performed to enhance signal sensitivity, ensuring that the generation of oxide ions and doubly charged species remained below 3% and 5%, respectively.

The CyTOF experiments were conducted in the XT Model (Standard BioTools, San Francisco, CA, USA) using a constant sample flow of 10 µL min^{-1}. Nucleic acid concentrations were determined by spectrophotometry using a NanoDrop instrument (Thermo Fisher Scientific, Bremen, Germany). Nanoparticle imaging was carried out with a JEM-2100F transmission electron microscope (TEM) from JEOL (Tokyo, Japan).

Materials and Methods

All solutions were prepared in ultrapure water from a PURELAB flex 3 from ELGA (High Wycombe, United Kindgom). When handling miRNA samples, DEPC-treated water (Ambion, Austin, TX, USA) was employed to ensure RNA integrity. To reduce nonspecific RNA adsorption to plastic ware, low protein-binding microcentrifuge tubes (Thermo Fisher Scientific) were utilized. RNAse contamination was prevented by cleaning all surfaces and materials with RNAseZap (Thermo Fisher Scientific).

Characterization and conjugation of PEGylated gold nanoparticles to DNA sequences.

Gold nanoparticles were 40 nm gold nanospheres coated with polyethylene glycol from CD Bioparticles (New York, USA). The characterization was done by TEM and by single particle ICP-MS as previously described.[1]

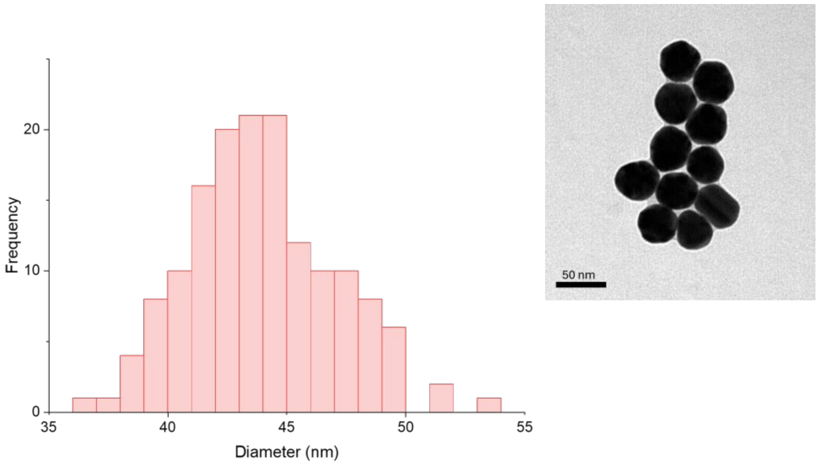

Figure S1. Characterization of the Au-nanoparticles used for the study by TEM.

Custom DNA oligonucleotides were synthesized by Invitrogen (Massachusetts, USA) and supplied in lyophilized form. They were reconstituted following the manufacturer's recommendations. Three specific sequences were employed in this work: (1) the target sequence corresponding to miR-16-5p, (2) a biotinylated DNA strand partially complementary to the 3' end of the miRNA (capture probe), and (3) another biotinylated strand complementary to the 5' end (detection probe). To circumvent degradation issues commonly associated with RNA handling, all optimization procedures were conducted using the DNA analogue of miR-16-5p, taking advantage of the efficient hybridization between RNA and DNA. Consequently, even in experiments involving actual miRNA samples, the capture and detection probes remained as DNA. Both complementary strands were

extended with poly(A) tails (21 adenines in seven AAA repeats for the capturer probe and 24 adenines in eight AAA repeats for the detection probe) to spatially separate the hybridization region from the biotin label, thereby reducing potential steric hindrance.

Table S1. Sequences used for the proposed strategy.

NAME	SEQUENCE
(1) miR-16-5p (target)	5'- UAG CAG CAC GUA AAU AUU GGC G -3'
(1)* miR-16-5p (surrogate DNA target)	5'- TAG CAG CAC GTA AAT ATT GGC G -3'
(2) Capture oligo (biotinylated)	5'- T TTA TAA CCG CAA $(AAA)_7$ -BIOT -3'
(3) Detection oligo (biotinylated)	5'- BIOT-$(AAA)_8$ ATC GTC GTG CA -3'

The detection probe was obtained by functionalizing 40-nm streptavidin-coated gold nanoparticles with the biotinylated detection oligonucleotide. To do this, 250 µL of the nanoparticle suspension was centrifuged at 10,000 rpm for 5 min to pellet the particles, the supernatant was discarded, and the pellet was re-suspended in Tris-buffered saline (TBS) buffer supplemented with 0.01% Tween 20. This solution was then combined with 16.7 µL of the biotinylated oligo and incubated at room temperature (RT) for 30 min. The resulting conjugates were purified by centrifugation at 10,000 rpm for 5 min, re-suspended in 500 µL of TBS, and subjected to a total of three washing cycles, which was established as the optimal number for probe separation.

Preparation of the capture probe

The capture probe was generated by linking the biotinylated capture oligonucleotide to streptavidin-coated magnetic microparticles. To begin, 4 µL of the magnetic beads were subjected to three washing steps using a magnetic separator and a buffer composed of 2 M NaCl, 1 mM EDTA, and 10 mM Tris in ultrapure water, adjusted to pH 7.5. Following this, 92 pmol of the oligonucleotide were added and incubated with the beads at RT for 20 min to allow for conjugation. Unbound oligonucleotides were subsequently removed through two additional washing cycles, using the magnet to retain the functionalized microparticles.

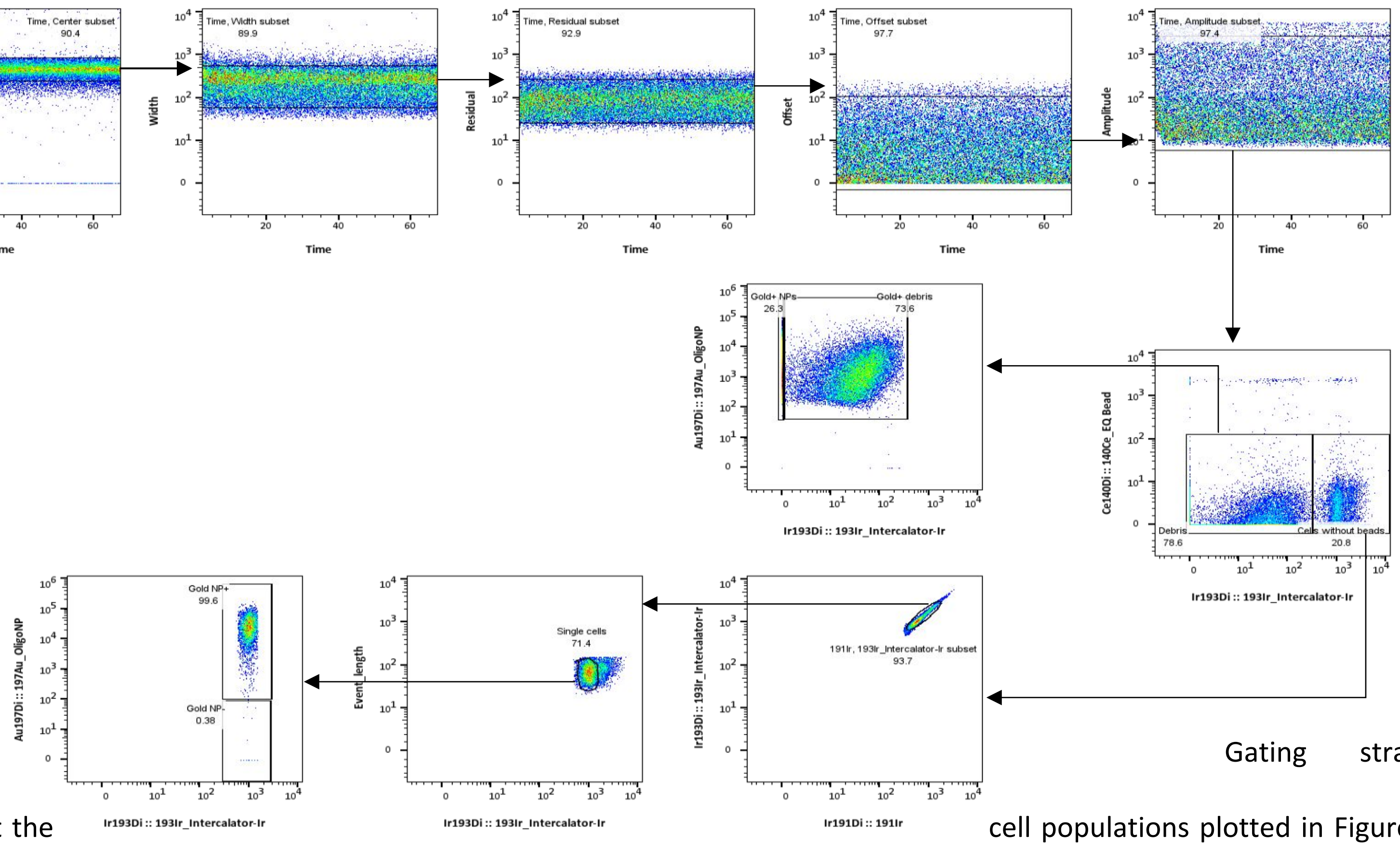

Figure S2. Gating strategy applied to select the cell populations plotted in Figure 2B, C, D and E regarding the mass cytometry-derived data.

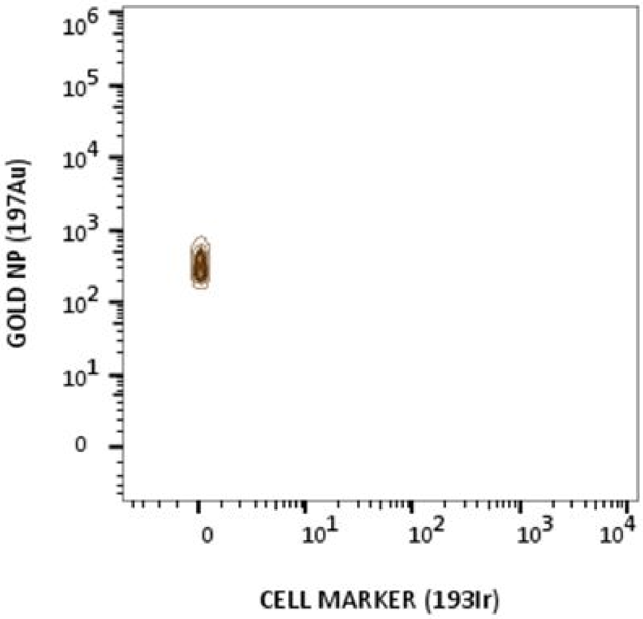

Figure S3. Obtained results of the suspension of gold nanoprobes analyzed using CyTOF.

Single Particle ICP-MS Measurement

Single particle ICP-MS analysis (SP-ICP-MS) with a TQ system was used as a tool for counting the gold nanoparticles after the assay, where the intensity of individual events was related to the gold mass per nanoparticle by means of a calibration curve constructed using elemental gold standards. This calibration took into account the instrument's transport efficiency, sample flow rate, and dwell time. Transport efficiency was determined daily using the particle number method and a quality control material of 30 nm gold nanoparticles (LGCQC5050, LGC, Teddington, UK), which has a concentration of 1.47 x 10^{11} particles g^{-1}. The suspension was diluted to a final concentration of 3.0 x 10^4 particles mL^{-1} in ultrapure water prior to use.

Under the conditions employed, typical transport efficiency values were approximately 5%, consistent with the use of a conventional sample introduction system composed of a MicroMist nebulizer and a cyclonic spray chamber. The sample flow rate was determined gravimetrically on each measurement day, typically yielding values close to 0.4 mL min^{-1}. Unless otherwise specified, data acquisition was conducted over a 2-minute interval, with a dwell time of 5 ms. Consequently, all reported event counts correspond to this acquisition time.

For nanoparticle quantification via SP-ICP-MS following the assay, data processing was carried out using a custom Microsoft Excel spreadsheet. This tool identified nanoparticle events by applying a threshold defined as the background signal plus three times its standard deviation, using an iterative method.[2,3] The number of detected events was then used to calculate the concentration of the target miRNA in the sample.

SP-ICP-MS for miRNA Determination Global Assay

A very similar procedure was carried out for the detection of miRNAs in the RNA extracted from the different cell lines. 100 µL of the extracted RNA is diluted to 500 µL in TBS. 26 µL and 141 µL of the freshly prepared detection and capture probes, respectively are added and incubated at 70 °C for 10 min. The mixture was then slowly cooled down to room temperature for 3 hours, washed twice with 500 µL of TBS, twice with 300 µL of TBS, and finally resuspended in 300 µL of TBS. An additional 10-minute incubation at 97ºC is needed in order to denature all nucleic acid hybrids and disrupt biotin-streptavidin interactions, releasing the gold nanoparticles from the magnetic beads. Subsequent magnetic separation

and appropriate dilution of the gold nanoparticle suspension was followed by analysis using SP-ICP-MS.

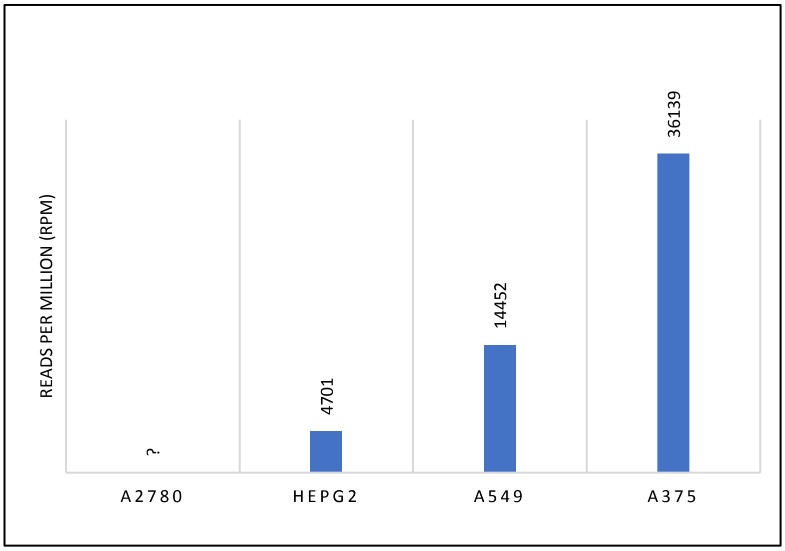

Figure S4. Levels of expression of miRNA16-5p (reads per million, RPM) in four different cancer cell lines according to reference 23 of the manuscript.

References

(1) González Morales, S.; López-Portugués, C.; Fernández-Sanjurjo, M.; Iglesias-Gutiérrez, E.; Montes Bayón, M.; Corte-Rodríguez, M. Amplification-Free Strategy for MiRNA Quantification in Human Serum Using Single Particle ICP–MS and Gold Nanoparticles as Labels. *Anal. Chem.* **2024**. https://doi.org/10.1021/acs.analchem.4c01904.

(2) Pace, H. E.; Rogers, N. J.; Jarolimek, C.; Coleman, V. A.; Higgins, C. P.; Ranville, J. F. Determining Transport Efficiency for the Purpose of Counting and Sizing Nanoparticles via Single Particle Inductively Coupled Plasma Mass Spectrometry. *Anal. Chem.* **2012**, *84* (10), 4633. https://doi.org/10.1021/ac300942m.

(3) Laborda, F.; Jiménez-Lamana, J.; Bolea, E.; Castillo, J. R. Selective Identification, Characterization and Determination of Dissolved Silver(i) and Silver Nanoparticles Based on Single Particle Detection by Inductively Coupled Plasma Mass Spectrometry. *J. Anal. At. Spectrom.* **2011**, *26* (7), 1362–1371. https://doi.org/10.1039/c0ja00098a.
